# Peer Review of “Using Structural Equation Modelling in Routine Clinical Data on Diabetes and Depression: Observational Cohort Study”

**DOI:** 10.2196/38488

**Published:** 2022-04-27

**Keywords:** depression, diabetes, electronic health records, acute care, PLS-SEM, path analysis, equation modelling, accident, emergency care, emergency, structural equation modelling, clinical data

*This is a peer-review report submitted for the paper “Using Structural Equation Modelling in Routine Clinical Data on Diabetes and Depression: Observational Cohort Study.”*

## Round 1 Review

### Major Comments

1. In this paper [1], the general research hypothesis should be interpreted and clarified more in the Introduction.
2. Please redesign Figure 1 with better quality and interpretations.
3. Recommendations and limitations are absent.

### Minor Comments

1. Order keywords alphabetically.
